# Transarterial Coil Embolization of a Symptomatic Posttraumatic Plantar Pseudoaneurysm

**DOI:** 10.1155/2015/453657

**Published:** 2015-03-19

**Authors:** Lukas Philipp Beyer, Walter A. Wohlgemuth, René Müller-Wille

**Affiliations:** Department of Radiology, University Medical Center Regensburg, Franz-Josef-Strauß-Allee 11, 93042 Regensburg, Germany

## Abstract

Posttraumatic pseudoaneurysms of the lateral plantar artery are rare. We report the case of a 31-year-old woman with a painful pseudoaneurysm of the lateral plantar artery resulting from a deep plantar cut injury. The pseudoaneurysm was successfully treated by performing a transarterial “frontdoor-backdoor” coil embolization technique, which is a minimally invasive alternative to conventional ligature of the artery.

## 1. Introduction

A posttraumatic pseudoaneurysm of the lateral plantar artery is a very rare but usually painful clinical entity. We report successful treatment of a symptomatic plantar pseudoaneurysm using endovascular coil embolization.

## 2. Case Report

A 31-year-old woman presented in an external hospital with a deep puncture wound on the sole of her left foot sustained by stepping on a sharp object. Due to heavy bleeding the wound was cleansed and closed using skin glue. A compression bandage was applied. Despite the treatment the woman complained of severe pain and had to rely on crutches. One month after the injury a phlegmonous inflammation developed at the site of the wound, which was surgically treated in the same hospital. Before and after this surgery a strong pulsation was palpable at the sole of the foot. 8 weeks later the woman presented in our outpatient department with persisting pain and severely restricted mobility. A pulsatile mass was palpable below the 3 cm long irritation-free scar on the sole of her left foot. Furthermore, dilated veins were present on the medial edge of the foot, the medial ankle, and the medial distal lower leg.

**Figure 1:**
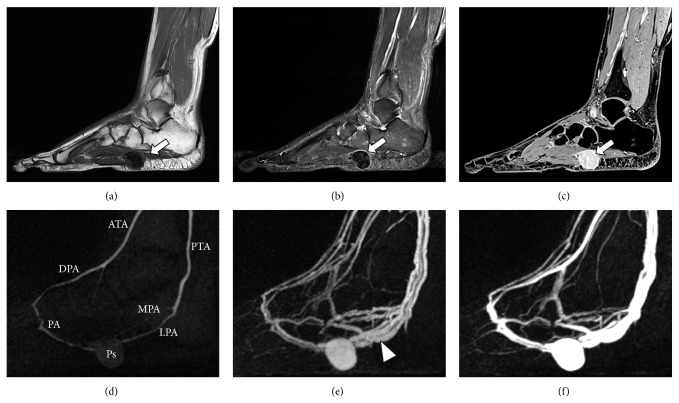
MRI and MRA two months after the trauma (3T Magnetom Skyra, Siemens AG Healthcare, Erlangen, Germany). (a) T1 image. (b) STIR (short-Ti inversion-recovery) image. (c) Gadolinium-enhanced VIBE (volumetric interpolated breath-hold examination) image using 6 mL Gadovist (Bayer Schering, Berlin, Germany). (d)–(f) TWIST (time-resolved angiography with interleaved stochastic trajectories) 3D MRA with a temporal resolution of 5 seconds. White arrow = pseudoaneurysm. White arrowhead = early filling of the veins due to arteriovenous fistula. ATA = anterior tibial artery. DPA = dorsalis pedis artery. PA = plantar arch. MPA = medial plantar artery. LPA = lateral plantar artery. PTA = posterior tibial artery. Ps = pseudoaneurysm of the lateral plantar artery.

Duplex sonography and an MRI scan of the left foot were subsequently carried out and revealed a 1.6 × 2.3 × 2.3 cm large pseudoaneurysm of the lateral plantar artery with a small arteriovenous fistula (Figure 1). The MRI scan was performed to confirm the diagnosis and rule out other causes of the symptoms. After interdisciplinary discussion of the various treatment options with the patient, we decided to undergo a minimally invasive coil embolization of the pseudoaneurysm.

**Figure 2:**
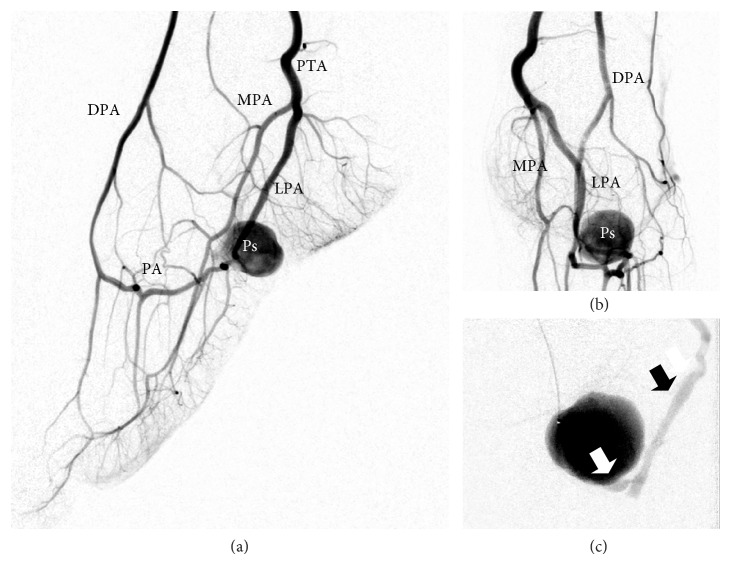
Diagnostic angiography through the 4-Fr catheter in the popliteal artery ((a), (b)), respectively, the microcatheter in the plantar arch (c). White arrow = arteriovenous fistula. Black arrow = early filling of a dilated vein. DPA = dorsalis pedis artery. PA = plantar arch. MPA = medial plantar artery. LPA = lateral plantar artery. PTA = posterior tibial artery. Ps = pseudoaneurysm.

**Figure 3:**
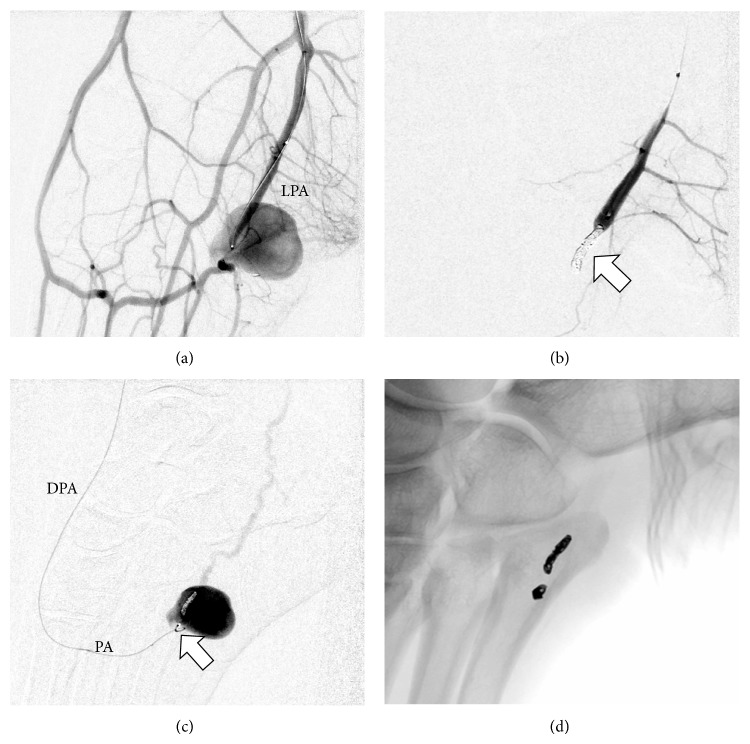
Frontdoor ((a) and (b)) and backdoor ((c) and (d)) coil embolization of the pseudoaneurysm with microcoils (white arrows). Angiography was performed through the 4-Fr catheter in the popliteal artery (a), respectively, the microcatheter in the lateral plantar artery (b) and plantar arch (c). The left lateral plantar artery was catheterized via the posterior tibial artery and the deep plantar arch via the dorsalis pedis artery (approached through the anterior tibial artery). DPA = dorsalis pedis artery. PA = plantar arch. LPA = lateral plantar artery.

**Figure 4:**
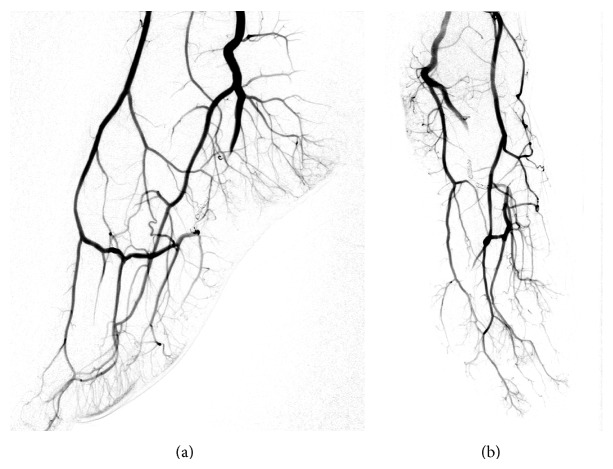
Final angiography confirming a complete occlusion of the pseudoaneurysm.

Arterial access was obtained by an antegrade puncture of the left common femoral artery using a 5-Fr sheath (Radifocus Introducer II, Terumo Corporation, Tokyo, Japan). A 4-Fr diagnostic catheter (Glidecath, Terumo, Tokyo, Japan) was placed in the P3 segment of the popliteal artery. The diagnostic angiography confirmed the MRA scan findings and demonstrated a pseudoaneurysm of the lateral plantar artery shortly before the transition to the deep plantar arch (Figure 2). Due to strong collateral circulation from the dorsalis pedis artery to the deep plantar arch we decided to secure the aneurysm using a “frontdoor-backdoor” coil embolization technique. After selective catheterization of the left lateral plantar artery via the posterior tibial artery using a microcatheter (Excelsior 10, Boston Scientific, Fremont, CA, USA) and a microwire (Synchro 10, Stryker Neurovascular, Fremont, CA, USA) the vessel was occluded with two electrolytically detachable microcoils (MicroPlex 10, 3 mm/8 cm and 2 mm/8 cm, MicroVention Inc., Aliso Viejo, CA) in proximity to the pseudoaneurysm (Figure 3). This was followed by a selective catheterization of the deep plantar arch via the dorsalis pedis artery (approached through the anterior tibial artery) and placement of two more microcoils (MicroPlex 10, 2 mm/6 cm and 2 mm/8 cm, MicroVention Inc., Aliso Viejo, CA) distal to the pseudoaneurysm. The final angiography demonstrated a complete elimination of the pseudoaneurysm (Figure 4) and the small arteriovenous fistula. All of the plantar metatarsal arteries showed a regular perfusion after the intervention.

The intra- and postinterventional course was free of complications and the patient was discharged on day 2 after the intervention. The pulsation was not palpable anymore after the intervention and no further imaging was necessary. After a temporary period of increased pain for about 1 week, which may have been caused by remodelling processes due to thrombosis of the aneurysm, the pain gradually decreased. She is now fully recovered and still pain-free after 6 months.

## 3. Discussion

Pseudoaneurysms of the plantar arch occur as a sequel of surgery [1, 2] or trauma, usually a deep cut injury [3–6]. The lateral plantar artery is presumed to be much more frequently affected than the medial plantar artery, possibly due to its larger diameter and more superficial location [5].

Severe pain and a palpable pulsatile mass are typical symptoms of a plantar pseudoaneurysm. The pulsatile blood flow and absence of a vascular wall cause pseudoaneurysms to increase gradually and eventually rupture weeks after the trauma [3]. The space-occupying effect of a plantar pseudoaneurysm can also cause damage to the adjacent nerves resulting in a tarsal tunnel syndrome [6].

Imaging techniques like duplex sonography [4], angiography [3], and MRI, in particular time resolved 3D MR angiography [7], are useful diagnostic tools. In the majority of cases they show a mass next to the sole of the foot which is perfused by a plantar artery.

Due to the extremely strong collateralization of the plantar arch, the proximal and distal site of the pseudoaneurysm can be occluded. Therefore surgical treatment consists not only of the excision of the pseudoaneurysm but also ligature of the artery on both sides [3, 5]. In our case the endovascular elimination of the pseudoaneurysm was realized by performing a “frontdoor-backdoor” coil embolization technique. We believe the main advantages of endovascular treatment compared to surgical ligation are faster recovery times, better protection of the surrounding tissue, and elimination of the pain and trauma associated with a plantar incision. We decided against coiling the pseudoaneurysm itself because we wanted to reduce the volume of the aneurysm to minimize the mass effect and associated risk of tarsal tunnel syndrome. Ultrasound guided percutaneous thrombin injection may also be performed but an arteriovenous fistula, as in the present case, is a contraindication. Thrombin could enter the venous circulation and may lead to distant thrombosis [8].

Possible complications of the “frontdoor-backdoor” coil embolization include those caused by femoral artery puncture (e.g., haematoma, arteriovenous fistula) and by contrast media (e.g., allergic reaction, nephropathy). Insufficient collateral circulation may lead to soft tissue necrosis.

Only two cases of plantar pseudoaneurysm treatment by coil embolization have been reported so far [6]. The first case was a 70-year-old man with a large pseudoaneurysm of the medial plantar artery acquired after a motorcycle accident that caused a medial cuneiform fracture (among other injuries). The second case was a 45-year-old man with a pseudoaneurysm of the medial plantar artery resulting from a laceration on his right foot sole sustained after hitting glass. Both patients suffered from tarsal tunnel syndrome triggered by the space-occupying effect of the pseudoaneurysms and were treated successfully using coil embolization.

We consider early diagnosis and early treatment to be important because of the risk of rupture and tarsal tunnel syndrome. In our opinion the main advantage of endovascular treatment over surgical treatment is the optimal protection of the surrounding tissue, especially the surrounding nerves. We believe “frontdoor-backdoor” coil embolization is superior to coiling the aneurysm itself because of volume reduction. We also believe an excision of the pseudoaneurysm after successful embolization is not necessary.
